# JAK2 mutational status and the contribution of TERT and JAK2 polymorphisms to the occurrence of myeloproliferative neoplasms in Eastern Morocco

**DOI:** 10.4314/ahs.v24i3.18

**Published:** 2024-09

**Authors:** Karam Yahya Belmokhtar, Mounia Elidrissi Errahhali, Saida Lhousni, Manal Elidrissi Errahhali, Rachida Bouagaga, Meryem Ouarzane, Majida Charif, Nadia Al Attar, Zaina Sidqi, Siham Hamaz, Houda Bachir, Khalid Andaloussi Serraj, Redouane Boulouiz, Habiba Alaoui, Mohammed Bellaoui

**Affiliations:** 1 Genetics Unit, Medical Sciences Research Laboratory, Faculty of Medicine and Pharmacy, University Mohammed Premier, Oujda, Morocco; 2 Department of Internal Medicine, Mohammed VI University Hospital, Laboratory of Immunohematology and Cellular Therapy, Faculty of Medicine and Pharmacy, University Mohammed Premier, Oujda, Morocco; 3 El Farabi Regional Hospital, Oujda, Morocco; 4 Transfusion Regional Centre, Oujda, Morocco

**Keywords:** Myeloproliferative neoplasms, JAK2 V617F, TERT and JAK2 polymorphisms, genetic predisposition, Morocco

## Abstract

**Background:**

The JAK2 V617F somatic mutation is a hallmark of myeloproliferative neoplasms (MPN) and is present in some patients with splanchnic venous thrombosis (SVT).

**Objectives:**

We investigated for the first time in Eastern Morocco the JAK2 mutational status and germline risk factors, such as the TERT and JAK2 polymorphisms, in MPN and SVT patients.

**Methods:**

This study included 38 patients with MPN, 24 patients presenting with SVT and 60 healthy donors from the BRO Biobank. JAK2 mutations were analyzed using qPCR and Sanger sequencing. Predisposing polymorphisms to MPN were evaluated using Sanger sequencing.

**Results:**

JAK2 V617F mutation was positive in 64.5% of patients with MPN and 20.8% of patients with SVT. The JAK2 V617F allelic burden ranged from 2% to 97.53%. We found a strong association between the JAK2 rs56241661 polymorphism of the JAK2 46/1 haplotype and the development of MPN. However, no association was detected between the TERT rs2736100 polymorpism and MPN.

**Conclusion:**

The JAK2 mutational status and its allelic burden in Eastern Morocco are consistent with previous studies. The JAK2 46/1 haplotype was strongly associated with MPN. However, unlike other previously studied populations, the TERT polymorphism rs2736100 has no effect on the occurrence of MPN in our population.

## Introduction

Myeloproliferative neoplasms (MPN) are hematological malignancies characterized by abnormal proliferation of one or more myeloid lineages. According to the revised 2016 World Health Organization (WHO) classification, MPN includes Chronic myeloid leukemia (CML), Polycythemia Vera (PV), Essential Thrombocythemia (ET), Primary Myelofibrosis (PMF), Chronic Eosinophilic Leukemia (CEL), Chronic Neutrophilic Leukemia (CNL) and MPN Unclassifiable (MPN-U) 1.

CML is characterized by the presence of a translocation between chromosomes 9 and 22 (Philadelphia chromosome (Ph))^2^. Most of the Philadelphia chromosome negative MPN (Ph-MPN) are characterized by the presence of driver mutations in genes such as Janus Kinase 2 (JAK2), Calreticulin (CALR), and Myeloproliferative Leukemia virus oncogene (MPL) 3. The JAK2 V617F mutation located in exon 14 is the most common driver mutation of these diseases. This mutation causes constitutive activation of the tyrosine kinase resulting in activation of STAT, PI3K and MAPK pathways inducing cytokine hypersensitivity 4. JAK2 V617F mutation is found in majority of PV patients (95%), 60% of ET and 50% PMF patients 5, 6. In addition to JAK2 V617F, several gain-of-function mutations in exon 12 have been described in about one third of JAK2-negative PV 7. Testing for JAK2 mutations is now a major diagnostic criterion for MPN^1^. It is also part of the diagnostic workup in other diseases such as splanchnic veins thrombosis (SVT), which includes the portal vein thrombosis (PVT), mesenteric splenic vein thrombosis (MVT), and the Budd Chiari syndrome (BCS). In fact, latent MPN are the main factor for the development of recurrent SVT, and are also the common etiology of PVT, MVT and BCS 8 9.

GWAS studies have identified a number of polymorphisms that increase the risk of developing sporadic MPN 10, 11. Among these predisposing polymorphisms, the JAK2 46/1 haplotype was the first major germinal risk factor identified in Caucasian population to predispose to the development of MPN, especially to JAK2 mutated MPN (V617F and exon 12 mutations) 12, 13. Several reports have confirmed the association between JAK2 46/1 haplotype and MPN in Chinese and Japanese populations, suggesting that the underlying mechanism is not limited to Caucasians population 14, 15. Other polymorphisms in the TERT gene which encodes a telomerase reverse transcriptase, such as rs2736100, rs2853677 and rs7705526, were also identified to predispose to the development of MPN 10, 11.

We have previously shown that MPN are the most common myeloid neoplasms in Eastern Morocco 16, 17. We have also shown that gene mutations analysis was carried out in only a small number of patients with MPN 16, 17. Here we investigated for the first time in Eastern Morocco the JAK2 mutational status and its allelic burden. Additionally, we examined the contribution of JAK2 and TERT polymorphisms to the occurrence of MPN in the East Moroccan population.

## Materials and Methods

### Patients and Controls

This study included 38 patients with clinically diagnosed MPN and 24 patients presenting with SVT recruited in the BRO-Biobank between 2018 and 2022. These patients were addressed to the BRO Biobank by the Department of Internal Medicine of Mohammed VI University Hospital and the Transfusion Regional Centre of Oujda (TRCO) at Oujda, Morocco. After reviewing the available medical records and according to the 2016 World Health Organization (WHO) guidelines the 38 MPN cases were classified as follows: 15 PV, 14 ET, 5 PMF and 4 MPN-U. The 24 cases with SVT were distributed as follows: 19 PVT, 3 MVT and 2 BCS. Clinical features of MPN and SVT patients are shown in Supplementary Tables 1 and 2. To assess the association between germline polymorphisms and the development of MPN, we compared 35 MPN patients with 60 sex matched healthy controls from the BRO Biobank. The control group had a median age of 34.5 years, with normal complete blood cell count and no history of malignancy.

**Table 1:** JAK2 V617F mutation positivity and allele burden by pathology

Pathology	*JAK2* V617F mutation positivity (%)^a^	*p*-value^c^	*JAK2* V617F allele burden (%)^b^	*p*-value^d^
SVT (n=24)	20.80%	<0.005	34.50% ± 23.50	NS
MPN (n=38)	60.50%		56.20%± 27.90	

ET (n=14)	64.30%	0.01	39.40% ± 15.90	0.01
PV (n=15)	66.70%		58.30%± 29.70	
PMF (n=5)	0%		-	
MPN-U (n=4)	100%		89.00% ± 8.64	

MPN: Myeloproliferative neoplasms, SVT: Splanchnic veinous thrombosis, PV: Polycythemia vera, ET: Essential thrombocythemia, PMF: Primary myelofibrosis, MPN-U: unclassifiable Myeloproliferative neoplasms

a: Number of *JAK2* V617F positive patients/total number of patients (%).

b: fraction of mutated alleles in *JAK2* V617F positive patients (mean).

c: *p* value obtained using Fisher's exact test.

d: *p* value obtained using Krustal-Wallis test.

**Table 2:** Distribution of JAK2 rs56241661 and TERT rs2736100 variants in MPN patients and controls

	N	+/+	+/-	-/-	Frequency^a^	*p*-value^b^	OR (95% CI)
***JAK2* rs56241661**							
Controls	60	46 (77%)	13 (22%)	1 (1%)	0.12		
MPN	35	15 (43%)	6 (17%)	14 (40%)	0.49	<0.001	6.61 (3.23-13.5)
V617F positive MPN	23	8 (35%)	2 (9%)	13 (57%)	0.61	<0.001	10.9 (4.88-24.3)
V617F negative MPN	12	7 (58%)	4 (33%)	1 (8%)	0.25	NS	2.33 (0.80-6.81)
***TERT* rs2736100**							
Controls	60	7 (12%)	29 (48%)	24 (40%)	0.64		
MPN	35	8 (23%)	14 (40%)	13 (37%)	0.57	NS	0.74 (0.41-1.36)
V617F-positive MPN	23	6 (26%)	9 (39%)	8 (35%)	0.55	NS	0.66 (0.33-1.33)
V617F negative MPN	12	2 (17%)	5 (42%)	5 (42%)	0.62	NS	0.93 (0.38-2.30)

MPN: myeloproliferative neoplasms, +/+: wild type homozygous, +/-: heterozygous, mutated homozygous; NS: not significant.

a: for *JAK2* rs56241661, it is the frequency of the allele carrying the deletion, For the *TERT* rs2736100, it is the frequency of the G allele.

b: *p* value obtained using Fisher's exact test.

### Ethics approval and consent to participate

The current study was conducted in accordance with the principles of the Declaration of Helsinki, and received an ethical approval from the ethical committee of the Faculty of Medicine and Pharmacy of Casablanca under the number: 42/14. All participants gave their written informed consent.

### DNA Isolation and JAK2 V617F mutation analysis

Genomic DNA was isolated from blood samples using the phenol/chloroform method as described elsewhere^18^. The detection of JAK2 V617F mutation and the quantification of its allele burden were determined using Ipsogen® JAK2 RGQ PCR kit (Qiagen, Germany), following manufacturer's instructions. The reaction was carried out on the Rotor-Gene Q real-time cycler and the analysis was performed on Rotor Gene Q software (Qiagen, Germany).

### JAK2 exon 12 mutations detection

Sanger sequencing and high-resolution melting (HRM) analysis were used to detect mutations in JAK2 exon 12 in all JAK2 V617F-negative PV cases. For Sanger sequencing, JAK2 exon 12 was amplified by PCR with the following primers (F: 5′-CTCCTCTTTGGAGCAATTCA-3′; R: 5′-GAGAACTTGGGAGTTGCGATA-3′). PCR product was purified and sequenced using the BigDye® Terminator v3.1 Cycle Sequencing Kit and the ABI 3500 Genetic Analyser (Applied Biosystems-Thermofisher). Sequence analysis and alignment were performed using the Seqscape software version V 4.0 and the reference sequence NM.004972.4 (hg38)

For HRM, Real-time PCR on the Rotor-Gene Q real-time cycler (QIAGEN) was conducted using geneMAPTM JAK2 Exon 12 mutation screening kit to detect the following six common mutations in JAK2 gene exon 12: p.Ile540_Glu543, p.Arg541_Glu543del, p.Phe537_Lys539del, p.His358Gln, + p. Lys539del, p.Lys539Leu, p.Asn542_Glu543del. After amplification, HRM analysis was performed by detecting the fluorescent signal during a temperature rise of 0.1°C increments from 62°C to 88°C.

### JAK2 rs5641661 and TERT rs2736100 genotyping

**The JAK2 rs56241661 (JAK2:** c.1641+179_1641+183delTCTTA), a tagging polymorphism of the JAK2 46/1 haplotype located in intron 12 19, was genotyped by sequencing using the same primers used for JAK2 exon 12 mutations detection (see above). The TERT rs2736100 (TERT: c.1574-3777G>T) polymorphism was genotyped by sequencing using the following primers: F: 5′-GCTGTTTTCCCTGCTGACTT-3 and R:5′-ACGTTGCTGTCACTCACTGG-3′.

### Statistical analysis

Statistical analysis was performed using Jamovi software, version 2.3. Differences between groups of samples were assessed by using Fisher's exact test and Krustal-Wallis tests. Hardy-Weinberg equilibrium (HWE) was evaluated using Fisher's exact test of observed versus predicted genotype frequencies. Odds-ratio (OR) and 95% confidence interval (CI) were calculated to evaluate the association between polymorphisms and susceptibility to MPN. A p value < 0.05 was considered statistically significant.

## Results

### JAK2 V617F mutation and its allele burden

We first analyzed the JAK2 V617F mutation and its allele burden, which is the most common mutation in MPN patients. The results are shown in Table 1. The JAK2 V617F mutational status differed significantly between the two main groups MPN and SVT (p<0.005). In fact, this mutation was detected in 60.5% of patients with MPN, whereas it was detected in only 20.8% of patients with SVT. For MPN patients, the frequency of JAK2 V617F mutation was almost the same between PV (66.7%) and ET patients (64.3%). However, this mutation was not detected in the five patients with PMF. Interestingly, the JAK2 V617F mutation was found in all patients with MPN-U (Table 1).

**Figure 1:**
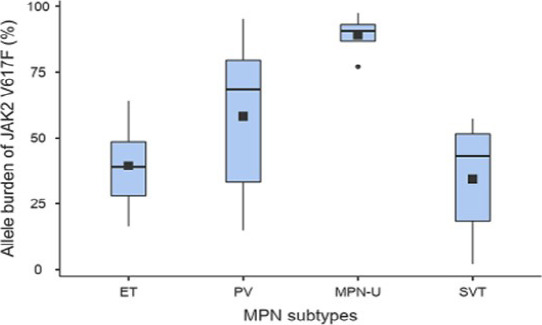
Comparison of the allele burden of *JAK2* V617F between MPN subtypes (ET=9, PV=10, MPN-U=4) and SVT (N=5). The squares represent the means and the horizontal line in the boxes represent the medians

Concerning JAK2 V617F-positive patients, the mean allele burden of JAK2 V617F differed significantly among MPN subtypes (p=0.01). Indeed, the mean allele burden of JAK2 V617F for ET (39.4% ± 15.9) was significantly lower than that for PV (58.3% ± 29.7) and MPN-U (89.00% ± 8.64) (Table 1). We noted that 70% of PV patients had an allelic burden above 50%. For ET patients, 77.7% had an allelic burden below 50%, whie all of MPN-U patients had an allelic burden above 75% (Figure 1). Regarding the patients with SVT, the mean allele burden of JAK2 V617F was 34.5% ± 23.5, and 60% of the patients had an allelic burden above 40%. It was 18.37% for the patient with BCS (1 positive case), 2% for the patient with MVT (1 positive case), and 50.65 ± 7.24% for the patients with PVT (3 positive cases).

### JAK2 exon 12 mutations

In addition to JAK2 V617F mutation, we screened for JAK2 exon 12 mutations in all JAK2 V617F-negative PV cases using two methods: a direct sequencing of the entire JAK2 gene exon 12 and a more senstive method (HRM) for the detection of 6 common mutations in JAK2 gene exon 12. For both analyses, no mutation was detected in all studied cases.

### Association of JAK2 rs5641661 and TERT rs2736100 polymorphisms in patients and controls

The JAK2 rs56241661 and the TERT rs2736100 polymorphisms were genotyped in 35 MPN cases and 60 controls (Table 2). The distribution of these two polymorphisms among controls were in HWE (p>0.05).

Regarding the JAK2 rs56241661 polymorphism, we found that 40% of MPN cases were mutated homozygous (-/-), 17% heterozygous (+/-) and 43% were wild type homozygous (+/+). In contrast, in the controls, 1% were -/-, 22% +/- and 77% +/+. Overall, the mutated homozygous genotype was significantly more frequent in MPN patients compared to the controls. This genotype distribution difference was also greater between JAK2 V617F-positive MPN and controls. We also found that the mutated allele frequency of this polymorphism was significantly higher in MPN (49%) and JAK2 V617F-positive MPN (61%) than in controls (12.5%). Association analysis revealed that this polymorphism was strongly associated with MPN (OR= 6.61, 95% CI = 3.23 - 13.5), especially with JAK2 V617F-positive MPN (OR = 10.9, 95% CI = 4.88 - 24.3). However, we found no association between this polymorphism and JAK2 V617F-negative MPN (Table 2).

For the TERT rs2736100 polymorphism, the frequency of the G allele was slightly lower in MPN (57%) and JAK2 V617F-positive MPN (55%) compared to the controls (64%), but this difference was not significant. Comparison of MPN, JAK2 V617F-positive MPN, JAK2 V617F-negative patients versus controls also revealed no difference in genotype distribution and no association between the TERT rs2736100 polymorphism and MPN or JAK2 V617F-positive MPN (Table 2).

## Discussion

### JAK2 V617F mutation and its allele burden

The discovery of the acquired JAK2 V617F mutation was a great breakthrough for the diagnosis of MPN 5, 6. Several reports of the JAK2 V617F mutation frequency in MPN showed that there is a wide variation, ranging from 51.6 to 79% in all MPN, from 79.2 to 100% in PV, from 25 to 70% in ET, from 12.5 to 90% in PMF and from 25 to 67.5% in MPN-U. In our study, the frequency of JAK2 V617F mutation in all MPN, PV, ET, PMF and MPN-U was 60.5%, 66.7%, 64.3%, 0% and 100% respectively. This result is in agreement with the frequencies reported for MPN subtypes in other studies 20-26, except for PV and PMF. Since we used highly sensitive assays here to detect JAK2 V617F mutation and given that all our patients have been classified according to the WHO diagnostic criteria, the low positivity rate observed in PV and PMF patients is most likely due to the small sample size. It is worth noting that for JAK2-negative patients, testing for MPL and CALR mutations is recommended in ET and PMF. Indeed, the discovery of mutations in these two genes has improved the accuracy of MPN diagnostic 27, 28. Regarding triple negative ET/PMF and JAK2-negative PV, it could be useful to consider a customized NGS panel or the whole exome sequencing approach to characterize the mutational landscape of these patients 29-31.

In this study, we found that 20.8% of the patients with SVT were JAK2 V617F-positive and that the mean allele burden was 34.10 ± 24.20 %. Such a finding is similar to those reported in other studies. Indeed, JAK2 V617F mutation has been detected in 17% to 21% of patients with PVT and MVT 9, and in 45.2% to 58.5% of patients with BCS 8. Thus, JAK2 V617F mutation screening is of high clinical usefulness for patients with SVT. Indeed, revelation of a latent MPN associated with SVT allows physicians to provide a prolonged anticoagulant treatment, especially if the blood count is normal 32.

Several studies suggested that the allele burden of JAK2 V617F is strongly associated with the different phenotypes of MPN and could be used as criterion for discriminating among the subtypes of MPN 33. Indeed, allele burden of JAK2 V617F is generally low in ET (< 25%), high in PV (> 50%), frequently high in PMF and close to 100% in post-PV/ET myelofibrosis 34. In this study, the lowest allele burdens were observed in ET patients and the highest allele burdens were observed in MPN-U. Furthermore, intermediate allele burdens were more frequent in PV. The higher allele burden in MPN-U suggests that this subtype could be an advanced stage of MPN.

Previous studies have shown that the allele burden of JAK2 V617F is generally low in patients with SVT. Indeed, How et al. (2018) reported a low allele burden (median of 5%, range: 0.06 to 9%) 35. Goodyer et al. (2016) also reported similar results, especially in patients with latent MPN (median of 9.6%, range 6.8-55.5%) 36. Patients with SVT have also distinct laboratory and clinical features such as younger age and high proportion of female 37. In this study, we also observed low to moderate allele burdens, a younger age (median age 47, range: 27-86), high proportion of female (62.5%), and subnormal laboratory values at diagnosis in patients with SVT. This trend towards a lower JAK2 V617F allele burdens and distinct clinical and laboratory characteristics suggests that patients with SVT may represent a distinct subtype of MPN or an early stage of the disease 38.

### JAK2 exon 12 mutations

The discovery of JAK2 exon 12 mutations has enriched the molecular diagnosis markers for JAK2 V617F-negative PV. Indeed, these mutations are associated with isolated erythrocytosis which explains their exclusive character in PV 7. Several reports showed a wide variation in frequencies of JAK2 exon 12 mutations, ranging from 2 to 20% in PV patients, depending on sample size and the sensitivity of the detection assays used 20, 26.

In this study, we used two techniques for the detection of JAK2 exon 12 mutations (Sanger sequencing and HRM assay), and in both conditions we did not detect any mutation in the JAK2 exon 12 sequence in all studied cases. This result is probably due to the small size of our sample, and therefore testing for JAK2 exon 12 mutations should be performed in a large cohort to better estimate their frequency in our PV patients.

### Contribution of JAK2 and TERT polymorphisms to the occurrence of MPN

Previous studies have identified several variants in different loci which have been associated with an increased risk of developing MPN in North American, European, Caucasian, Chinese and Japanese populations. Among these variants, the JAK2 46/1 haplotype was the first germinal major risk factor 12, 13, 19, 39.

Here, we found a strong association between the JAK2 46/1 haplotype and the occurrence of MPN, especially JAK2 V617F-positive MPN. Thus, this haplotype is also a germline risk factor in Moroccan population. It is worth noting that the JAK2 46/1 haplotype in our control population was found at a frequency of 12.5 %, which is similar to what was reported in African populations (15.87% in the ALFA allele frequency project), and significantly lower compared to what was found in Caucasian and Asian populations (22 to 29 %) 12-15, 19, 39-42. Further extensive study with a large control cohort is necessary to understand this difference in JAK2 46/1 haplotype frequency between the different populations and its impact on the occurrence of MPN.

In this study, we found no association between the TERT rs2736100 polymorphism and MPN, regardless of the JAK2 V617F mutational status, which is different from what has been found in other studies 43-45. This polymorphism was also associated with solid cancers (especially lung cancer and gliomas) which indicates that this polymorphism has probably a general genome effect 46. Further studies using a large age and sex-matched cohort are needed to confirm the lack of association between the TERT rs2736100 and MPN in our population.

## Conclusion

This study suggests that the JAK2 mutational status and its allelic burden in Eastern Morocco are consistent with previous studies. The JAK2 46/1 haplotype was strongly associated with MPN. However, unlike other previously studied populations, the TERT polymorphism rs2736100 has no effect on the occurrence of MPN in our population. Further studies are needed using a large cohort to better characterize MPN in our population and to identify other driver mutations and germline risk factors for MPN.
